# Cardiac Myosin Filaments are Maintained by Stochastic Protein Replacement

**DOI:** 10.1016/j.mcpro.2022.100274

**Published:** 2022-07-31

**Authors:** Neil B. Wood, Colleen M. Kelly, Thomas S. O’Leary, Jody L. Martin, Michael J. Previs

**Affiliations:** 1 Department of Molecular Physiology and Biophysics, University of Vermont, Larner College of Medicine, Burlington, Vermont, USA; 2 Department of Pharmacology, University of California, Davis, Davis, California, USA

**Keywords:** heart muscle thick filament, myosin-binding protein C, protein turnover, macromolecular complex, dynamic stable isotope labeling of amino acids in mammals, AAV, adeno-associated virus, AGC, automatic gain control, MyBP-C, myosin-binding protein C, RLC, myosin regulatory light chain

## Abstract

Myosin and myosin-binding protein C are exquisitely organized into giant filamentous macromolecular complexes within cardiac muscle sarcomeres, yet these proteins must be continually replaced to maintain contractile fidelity. The overall hypothesis that myosin filament structure is dynamic and allows for the stochastic replacement of individual components was tested *in vivo*, using a combination of mass spectrometry– and fluorescence-based proteomic techniques. Adult mice were fed a diet that marked all newly synthesized proteins with a stable isotope-labeled amino acid. The abundance of unlabeled and labeled proteins was quantified by high-resolution mass spectrometry over an 8-week period. The rates of change in the abundance of these proteins were well described by analytical models in which protein synthesis defined stoichiometry and protein degradation was governed by the stochastic selection of individual molecules. To test whether the whole myosin filaments or the individual components were selected for replacement, cardiac muscle was chemically skinned to remove the cellular membrane and myosin filaments were solubilized with ionic solutions. The composition of the filamentous and soluble fractions was quantified by mass spectrometry, and filament depolymerization was visualized by real-time fluorescence microscopy. Myosin molecules were preferentially extracted from ends of the filaments in the presence of the ionic solutions, and there was only a slight bias in the abundance of unlabeled molecules toward the innermost region on the myosin filaments. These data demonstrate for the first time that the newly synthesized myosin and myosin-binding protein C molecules are randomly mixed into preexisting thick filaments *in vivo* and the rate of mixing may not be equivalent along the length of the thick filament. These data collectively support a new model of cardiac myosin filament structure, with the filaments being dynamic macromolecular assemblies that allow for replacement of their components, rather than rigid bodies.

## Highlights

• A novel D_3_-leucine labeled diet enables mechanistic studies of protein turnover.
• Proteins are stochastically replaced within large macromolecular complexes.
• Replacement is facilitated by the exchange of free monomers in and out of complexes.
• Maintenance of a giant macromolecular complex is facilitated by dynamic exchange.

## Graphical Abstract

**Figure:**
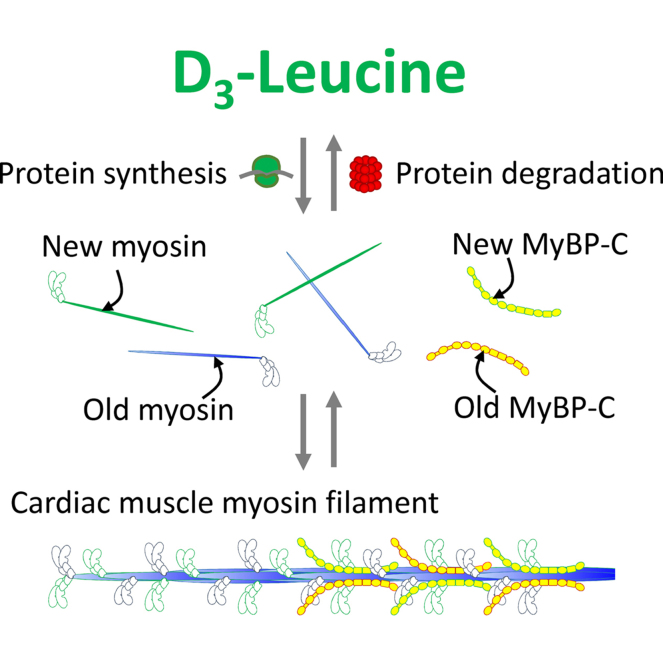

## In Brief

To examine the mechanisms of myosin filament replacement in mouse hearts, we labeled proteins with D_3_-leucine and a GFP-tag *in vivo*. We used a combination of high-resolution mass spectrometry, analytical modeling, biochemical fractionations, and fluorescence imaging to demonstrate that individual molecules are stochastically replaced from within these large macromolecular filamentous complexes.

**Fig. 1:**
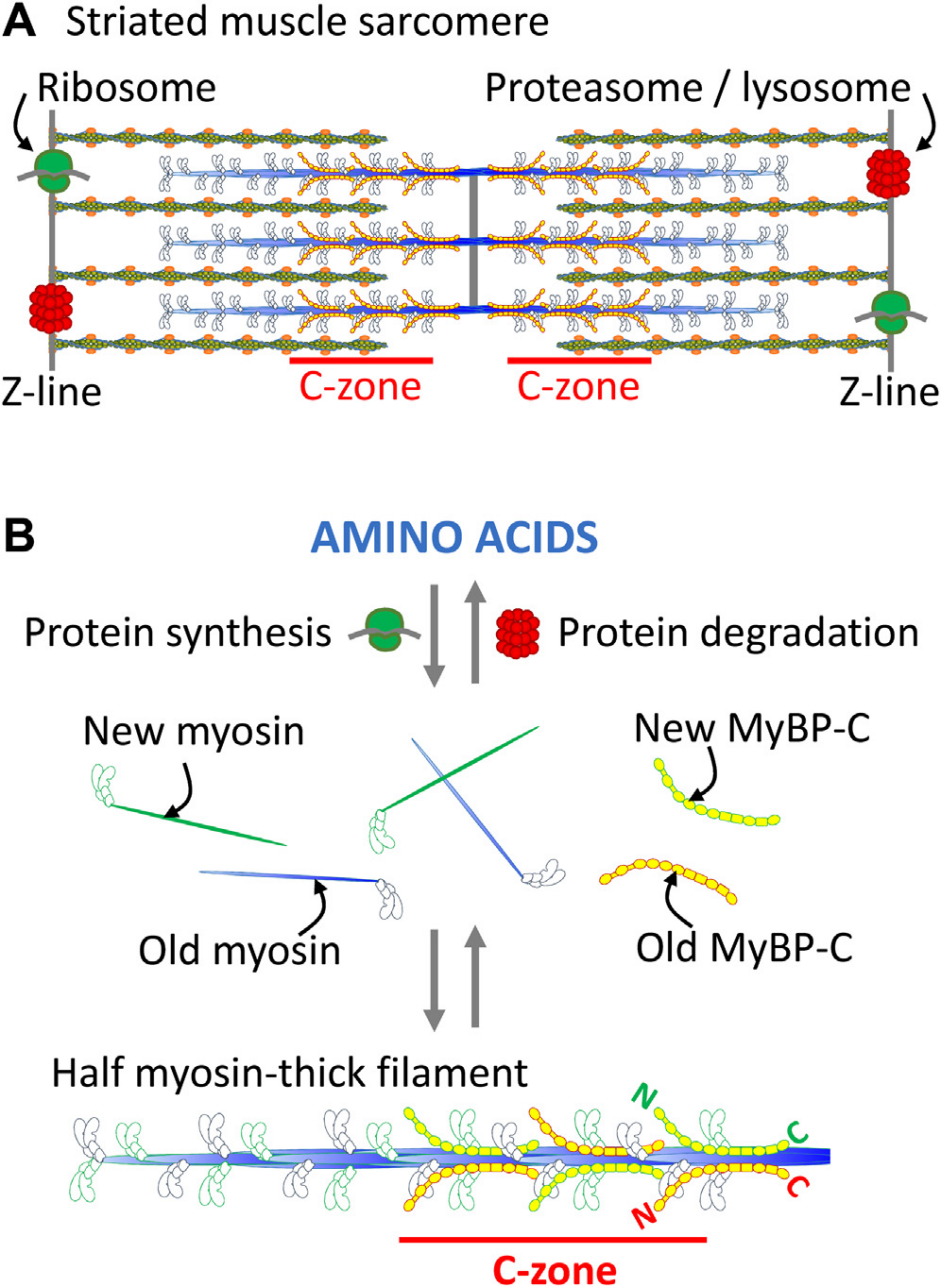
**Thick filament protein synthesis and degradation within a striated muscle sarcomere.***A*, illustrative representation of the localization of myosin-based thick filaments, actin-based thin filaments, ribosomes, and proteasomes/lysosomes within a single sarcomere. *B*, proposed model for the random exchange of myosin and MyBP-C molecules into and out of a *thick filament*.

Approximately 300 molecules of myosin and 54 molecules of myosin-binding protein C (MyBP-C) copolymerize to form each bipolar thick filament within a striated muscle sarcomere (Fig. 1*A*), being the large macromolecular assemblies that power muscle contractility. The quaternary structure of each myosin molecule (Fig. 1*B*) comprises two heavy chains. The N-terminal portion, or head of each heavy chain, is decorated with two light chains (one essential and one regulatory) that are critical for actomyosin force generation (1). The C-terminal portions, or tails of two heavy chains, dimerize *via* a coiled-coil to form a single myosin molecule (Fig. 1*B*). This coiled-coil is essential for the intermolecular ionic interactions between myosin molecules that form the backbone of the thick filament (Fig. 1*B*) (2). Myosin-binding protein C (MyBP-C) is a flexible rod-like protein comprising eight immunoglobulin and three fibronectin domains and two flexible linkers (Fig. 1*B*) that regulate its molecular function (3). The C-terminal domains of MyBP-C bind along the surface of the thick filament backbone (4, 5) and interact with super-repeats within titin, being a giant protein that spans the length of the sarcomere (6). These super-repeats within titin are located nearest the inner regions of each half-sarcomere and thus restrict the localization of MyBP-C to these regions, called the C-zones (Fig. 1*A*). Many details are known about the molecular structure and biophysical function of striated muscle thick filaments (4, 7, 8, 9) but there is a significant gap in our understanding of how these critical structures are maintained in an intact heart, which must continually beat throughout one’s lifetime.

The ribosomes, mRNA, proteasomes, and lysosomes required for the synthesis and degradation of the sarcomeric proteins are localized at the periphery, and/or Z-lines, of the fully developed sarcomere (Fig. 1*A*) (10, 11, 12, 13, 14, 15, 16). Developmental studies provide mechanistic insight into the initial formation of thick filaments within nascent sarcomeres (17, 18, 19, 20), and a recent report has demonstrated that new sarcomeres may be added to cells during gross cellular remodeling (21). However, tracking of the incorporation of labeled amino acids into newly synthesized proteins in cultured cells (22, 23) and animals (24, 25, 26, 27) demonstrates that myosin and MyBP-C are continually replaced on relatively rapid (*i.e.*, hour to day) timescales. Unlike skeletal muscle (28), the replacement of these proteins within the heart must come from within existing cells, because <1% of cardiac myocytes are turned over each year (29).

Early reports suggested that a population of thick filaments is easily released from muscle, leading to the hypotheses that the replacement of myosin and MyBP-C would involve the turnover of intact thick filaments and/or whole sarcomeres (30, 31, 32). However, neither partial sarcomeres nor dissociated thick filaments are observed in images of adult cardiac muscle tissue (33). In contrast, multiple lines of evidence support the alternative hypothesis that thick filament structure is dynamic and allows for the replacement of individual molecules (11). Seminal observations by Fischman and colleagues demonstrated that skeletal myosin molecules can exchange between preformed synthetic thick filaments *in vitro* (34, 35). Subsequent studies by Eisenberg/Russell and colleagues provided physiological support for this hypothesis by demonstrating the localization of myosin mRNA within the sarcomere (10). More recent studies have added fluorescent tags to myosin and MyBP-C *in vitro* and observed such exchange of molecules by fluorescence recovery after photobleaching (36, 37, 38). Yet it is not well accepted that thick filament structure is dynamic *in vivo*.

Advances in our ability to isotopically label the precursor pool of amino acids in animals, track the incorporation of these amino acids into newly synthesized proteins using mass spectrometry, and model these data have revolutionized our understanding of the mechanisms underlying protein turnover *in vivo* (39, 40, 41, 42). To test the hypothesis that the maintenance of cardiac muscle thick filaments involves the stochastic replacement of individual myosin and MyBP-C molecules from within these large macromolecular complexes, adult mice were fed a custom diet in which 99% of the leucine was replaced with isotopically labeled D_3_-leucine (where D is the stable isotope deuterium). The diet had no adverse impact on whole heart structure or molecular function as determined using an *in vitro* motility assay. The temporal and spatial dynamics of protein replacement were determined by a combination of high-resolution quantitative mass spectrometry, analytical modeling, biochemical extractions, and real-time fluorescence imaging assays. The data support a model in which the thick filament is viewed as a dynamic macromolecular assembly, built in such a way to allow for the stochastic replacement of its individual components, as required for intracellular maintenance.

## Experimental Procedures

### Mice

To examine the replacement of thick filament proteins in the hearts of adult mice, 24-week-old FVB mice were purchased from Charles River Laboratories and treated as described in the experimental procedures. In all procedures, mice were euthanized by anesthetization with isoflurane, cervical dislocation, and exsanguination by surgical removal of their hearts. Tissue was flash frozen in liquid nitrogen and stored in a -80 °C freezer until use, unless noted in the experimental procedure. All animal protocols were approved by the Institutional Animal Care and Use Committee at the University of Vermont and were in accordance with the guidelines listed in the Guide for the Use and Care of Laboratory Animals published by the National Institutes of Health.

### Isotopically Labeled Diet

To label newly synthesized proteins, a Mouse Express Kit was purchased from Cambridge Isotope Laboratories. The kit consists of a control diet and nutritionally identical diet in which 99% of the leucine molecules contained three deuterium atoms on the fifth carbon (5,5,5-^2^H_3_ L-leucine).

### Adeno-Associated Virus Design and Production

Mouse MLC2v (ventricular regulatory light chain [RLC]) was fused in frame with enhanced green fluorescent protein in pAAV-EGFP-miR155 containing the TnT promoter (courtesy of Dr Jiang Jianming, National University of Singapore) with the miR155 removed and sequenced for confirmation. To construct and purify AAV2/9.45, pAAV.cTnT.MLC2vGFP cDNA was cotransfected along with pAAV2/9.45 and pHelper (Stratagene) into adeno-associated virus (AAV)-293 cells. AAV stocks were titered for vector genomes by slot blotting (psoralen-biotin and Brightstar kits from Ambion), and titer was confirmed by sodium dodecyl sulfate gel analysis of capsid proteins. AAV titers were 1.5 × 10^13^ vector genomes/ml.

### Effect of the Diet on Body and Cardiac Muscle Mass

To ensure the mice were fully grown and determine whether Mouse Express affected body or heart mass, mice were weighed after cervical dislocation and cardiac mass was measured. The hearts were thawed in a phosphate-buffered saline solution, the atria were surgically removed, and the left and right ventricles were separated. Each piece of heart was weighed, flash frozen in liquid nitrogen, and stored at −80 °C for analyses. These data were separated by sex, plotted with respect to time using GraphPad Prism 8, and fitted with linear regressions to determine whether the slope of each line was significantly different than zero (*p* < 0.01 was considered significant). The difference in mass between males and females was determined by a Student’s *t* test.

### Effect of the Diet on Myosin Function

To determine whether the incorporation of the D_3_-leucine into myosin affected its molecular function, myosin was purified from three unlabeled (day 0) and three maximally labeled (day 56) hearts and its function was examined. Myosin monomers were purified from ∼30-mg pieces of each left ventricle by biochemical extraction and centrifugation, and actomyosin motility was determined in an *in vitro* motility assay, as described (43). Briefly, 100 μg/ml of monomeric myosin was adhered to a nitrocellulose-treated glass coverslip, and tetramethylrhodamine-phalloidin-labeled chicken skeletal actin filaments were distributed on the surface of the myosin molecules. The labeled actin filaments were visualized by epifluorescence on a custom-built Nikon-based microscope in the presence of 1 mM adenosine triphosphate (ATP) at 30 °C. Movies were recorded, and actin filament sliding velocities were determined using Manual Tracking/TrackMate in ImageJ (44). The mean velocity sliding velocities ±SEM for 119 filaments from the three biological replicates in each group were calculated. A Student’s *t* test was used to test for significance.

### Proteomics: Experimental Design and Statistical Rationale

The replacement of thick filament proteins in adult mouse hearts was examined by tracking the incorporation of labeled amino acids into the proteins within the hearts of 11 male and 11 female mice using mass spectrometry. The 24-week-old mice were fed unlabeled Mouse Express for 4 weeks, so they could adjust to the composition of the diet. On day 0, hearts were harvested from three mice to determine the natural distribution of stable isotopes, being control values. The feed for the remaining mice (n = 19) was replaced with D_3_-leucine Mouse Express, and the mice ate *ad libitum*. At days 5, 10, 18, 28, 42, and 56, 3 to 4 mice of mixed sex were euthanized and their hearts were collected, flash frozen in liquid nitrogen and stored in a −80 °C freezer. This provided three biological replicates for days 0 to 42 and four biological replicates for day 56. Proteomic experiments were performed to quantify the synthesis and degradation of the myosin filament proteins and determine their localization within the cardiac muscle. The hindlimbs, livers, and brains were flash frozen in liquid nitrogen and stored in a −80 °C freezer. For all experiments, data were fitted using GraphPad Prism 8, and significance between fits was determined as described under each experimental protocol.

## Quantification of the Synthesis and Degradation of myosin Filament Proteins by Mass Spectrometry

### Preparation of Samples for Mass Spectrometry

A 1- to 2-mg piece of the apex of each left ventricle was solubilized, the proteins were reduced, alkylated, and digested to peptides using trypsin as described (43). In brief, each piece of muscle was placed in a glass bottom dissection chamber containing 150 μl 0.1% Rapigest SF Surfactant (Waters) and mechanically triturated with forceps. The solubilized proteins were reduced by addition of 0.75 μl 1 M dithiothreitol (DTT), and heating at 100 °C for 10 min. Proteins were alkylated by addition of 22.5 μl of a 100 mM iodoacetamide (Acros Organics) in 50 mM ammonium bicarbonate, followed by a 30-min incubation in the dark at ∼22 °C. The proteins were cleaved into tryptic peptides by addition of 25 μl of 0.2 μg/μl trypsin (Promega) in 50 mM ammonium bicarbonate and incubation for 18 h at 37 °C. Following the digestion, the samples were dried down by centrifugal evaporation and reconstituted in 100 μl of a 7% formic acid in 50 mM ammonium bicarbonate solution to inactivate trypsin and degrade Rapigest (1 h, 37 °C). Samples were dried down once more and reconstituted in 100 μl 0.1% trifluoroacetic acid (TFA) for further cleavage of Rapigest (1 h, 37 °C). Samples were dried down a final time, reconstituted in 150 μl 0.1 TFA, centrifuged for 5 min at 18,800 RCF (Thermo, Sorvall Legend Micro 21R), and 125 μl of the supernatant was removed for analysis by mass spectrometry.

### Liquid Chromatography Mass Spectrometry

Tryptic peptides were separated *via* high-pressure liquid chromatography (LC) on an XSelect HSS T3 column (100 Å, 3.5 μm, 1 × 150 mm) (Waters Corporation) attached to an UltiMate 3000 ultra-high-pressure liquid chromatography system (Dionex). A 20-μL aliquot of each sample was injected into 0.1% formic acid in 2% acetonitrile with a flow rate of 100 μl/min. Starting at 2 min, the gradient was ramped linearly to 0.1% formic acid in 35% acetonitrile over 92 min. This was followed by another linear increase to 50% acetonitrile over 10 min. Finally, the gradient was ramped linearly to 0.1% formic acid in 90% acetonitrile over 4 min and held isocratic for 12 min. The column was then re-equilibrated over 27 min, prior to the next injection. The total run time was 145 min per injection.

The LC effluent was directly infused into a Q Exactive Hybrid Quadrupole-Orbitrap mass spectrometer (Thermo) through an electrospray ionization source. The instrument was operated in positive electrospray ionization mode with a capillary temperature of 320 °C and a spray voltage of 3.80 kV. Spectra were collected at a resolution of 70,000 while scanning from *m/z* = 200 − 2000 using automatic gain control (AGC). The AGC target was 3e6 with a max injection time of 100 ms. Data-dependent tandem mass spectrometry spectra were collected at a resolution of 17,500 with an AGC target of 1e5 and max injection time of 50 ms. The five most abundant peptides were selected for fragmentation using an isolation width of 1 *m/z* and normalized collision energy of 30. The minimum AGC target was 8e3 with an intensity threshold of 1.6e5 and a 10 to 20 s apex trigger activated. Masses were selected for fragmentation 5 times and then excluded for 15 s. Data were collected as Thermo Xcalibur .*raw* files.

### Mass Spectrometry Data Analysis Search Parameters

The .*raw* files were initially examined with an automated routine using Thermo Proteome Discoverer 2.2.0.388 (PD 2.2). The tandem mass spectrometry spectra were searched by Sequest HT against a *Mus musculus* database (containing 74,085 sequences, downloaded 02/09/15 from Uniprot). The database was digested *in silico* using trypsin, and up to two missed cleavages were permitted. The search parameters included carbamidomethyl as a static modification of cysteine. Variable modifications included oxidation (Met, Pro), phosphorylation (Arg, Ser, Thr, Tyr), and dioxidation (Met). In addition, the label:2H(3) (Leu) modification was included as a variable modification to identify peptides with one or more D_3_-leucine residues. The search used a precursor mass tolerance of 10 ppm and a fragment mass tolerance of 0.02 Da. A maximum delta correlation score of 0.05 was used for acceptance of peptide spectra. A maximum false discovery rate of 0.01 by decoy database search was applied to filter for high confidence peptides.

### Quantification of Peptide and Isotopomer Abundances

To quantify abundances of all peptides identified in the automated searches, areas under each LC peak were generated using the Precursor Ion Quantifier node in PD 2.2. The Minora Feature Detector was enabled to identify LC peaks with the exact mass, charge states, and elution time as the SEQUEST derived peptide spectral matches across the samples in the entire study. The resultant LC peak areas from these analyses were exported to Excel (supplemental Table S1) and summed.

To quantify the abundance of each isotopomer within peptides of interest, leucine-containing peptides identified by the automated searches were manually evaluated using Xcalibur Qual Browser software (Thermo). Full-MS scans within the region encompassing the chromatographic peak for the peptide of interest were averaged, noting that the heavy isotope-labeled species eluted slightly earlier than their unlabeled counterparts, as described (45). Thus, the averaged mass spectrum was created using a larger retention-time window than that necessary for any given mass isotopomer, to capture all isotopomers. To account for variation in the size of this retention-time window, the average intensity value for each m/z peak was multiplied by the number of mass spectrometry scans used to create the averaged spectrum. These resultant values were equivalent to integrated peak area for each mass isotopomer. The resultant LC peak areas for each mass isotopomer were exported to Excel (supplemental Table S2). These abundances were then normalized to the summed abundance of all peptides detected by PD 2.2, to account for any difference in the amount of starting material or analytical losses during sample processing and injection (46).

### Quantification of the Abundance of Unlabeled and D_3_-Leucine in the Precursor Pool

Although the diet contained 99% D_3_-leucine, we anticipated that the precursor pool of leucine is derived from a combination of leucine ingested from the diet and recycled from the breakdown of preexisting proteins, as observed for valine (40). Therefore, prior to quantifying thick filament protein turnover, we first needed to determine the abundance of unlabeled and D_3_-leucine within the precursor pool. This abundance was determined from the binomial distribution of D_3_-leucine in peptides within peptides containing 3 to 4 leucine residues using mass isotopomer distribution analysis (39, 40). First, the abundance of the naturally occurring isotopomers was subtracted from the isotopomer distribution. Next, the abundance of the D_3_-leucine in the precursor pool (expressed as a proportion, *r*) was determined from the ratio of the abundance of the labeled isotopomers at any given time point. An example of these calculations is shown for the ratio of the abundances of the isotopomers resulting from the incorporation of 2 (*I*_*2L*_) and 3 (*I*_*3L*_) D_3_-leucine molecules into a peptide with three potential sites of incorporation:

$$\frac{I_{3L}}{I_{2L}}=\frac{r^{3}}{3r^{2}\left(1-r\right)}$$

The abundance of the D_3_-leucine in the precursor pool (r) was then determined as follows:

$$r=\frac{3x}{3x+1}$$

where *x* is *I*_*3L*_/*I*_*2L*_. The same principle was used to derive *r* from the ratio of the abundances of the isotopomers resulting from the presence of 1 (*I*_*1L*_) and 2 (*I*_*2L*_) D_3_-leucine residues in the same peptide. The average abundance of *r* was determined by both approaches for five peptides from each heart sample. The average abundance of D_3_-leucine in the precursor pool for each mouse was plotted using GraphPad Prism 8 and fitted with a two-phase exponential equation.

### Quantification of the Abundance of Unlabeled and Deuterium-Labeled Proteins

Owing to the large change in enrichment in the precursor pool, the abundance of the unlabeled and labeled proteins was only determined from peptides containing two or more leucine residues. This maximized the probability that a newly synthesized protein contained at least one labeled residue. First, the abundances of the M + 0 through M + 2 isotopomers (*unlabeled peptides*) and M + 3 and all subsequent isotopomers (*deuterium-labeled peptides*) were summed separately. Next, the overlap of naturally occurring isotopes from *unlabeled peptides* into *deuterium-labeled peptides* was corrected using the natural distribution of isotopes measured from the three unlabeled (day 0) control samples. The probability that any *unlabeled peptide* selected was synthesized after the start of the labeled diet was <4.7 ± 3.6%, yet a second correction was applied to account for this phenomenon. The corrected abundance values for *unlabeled peptides* were indicative of the amount of protein translated prior to the start of the D_3_-leucine diet. The corrected abundance values of the *deuterium-labeled peptides* were indicative of protein translated after day 0 of the experimental protocol. We refer to these values as unlabeled and deuterium-labeled protein for simplicity throughout the article.

### Determination of the Turnover rates of the Unlabeled and Deuterium-Labeled Proteins

The abundances of the unlabeled and deuterium-labeled proteins in the hearts were determined with respect to time. All of the abundances were normalized to the summed abundance of all peptides detected by the automated PD analysis to account for any difference in the amount of starting material or analytical losses during sample processing and injection (46). For each protein of interest, the abundances of the unlabeled and deuterium-labeled peptides were normalized to the average abundance of the unlabeled peptide in the unlabeled samples (day 0) and then averaged. The average abundance of the unlabeled and labeled protein in each sample was plotted in GraphPad Prism 8 and fitted to a one phase exponential:

$$Abundance=\left(Abundance\;at\;t0-Plateau\right)*e^{-kt}+Plateau$$

where *t* is time in days, with the rate constant *k* and plateau being determined from the fit. Significant differences of rate constants and plateaus between abundance series were determined using an extra sum-of-squares F test.

## Assessment of the Localization of D_3_-Labeled Myosin Filament Proteins in Cardiac Muscle

### Quantification of Protein Solubility

To determine whether intact thick filaments or individual molecules of myosin and MyBP-C are extracted from muscle, 1- to 2-mg pieces of the left ventricle tissue were separated into soluble and filamentous fractions under a range of biochemical conditions. Protein abundances in each fraction were quantified by mass spectrometry. Tissue was taken from three mice fed a normal diet, providing three biological replicates for each condition. The tissue was mechanically triturated with forceps then rocked in a dissection chamber containing 400 μl of a relaxing solution containing KCl, 1 mM EGTA, 10 mM DTT, 25 mM imidazole, 4 mM MgCl_2_, adjusted to pH 7.4; with 0.4 mg/ml creatine phosphate, 0.7 mM adenosine triphosphate (ATP), 7 mM DTT, and 0.1% triton-X 100 detergent, with 33 μg/ml bovine serum albumin as an internal standard for quantification. The extraction times and KCl concentrations were varied as specified.

To determine the time it took for the tissue to equilibrate with relaxing solution containing 175 mM KCl, tissues were triturated and rocked for 5, 10, 20, 30, and 60 min. The tissues were transferred to microcentrifuge tubes, centrifuged at 18,800 RCF for 5 min (Thermo, Sorvall Legend Micro 21R), and the supernatants were placed in new microcentrifuge tubes. To determine the impact of ionic strength on protein solubility, the KCl concentration was varied (150, 175, 200, 225, and 250 mM). The tissues were triturated and rocked for 60 min prior to collection of the supernatants to ensure full equilibration with the solution. In these experiments a second aliquot of 400 μl of relaxing solution was added to the pellet for an additional extraction, but the abundances of the protein in this soluble fraction was combined with that in the pellet, for consistency between experiments. To determine whether the D_3_-labeled protein was preferentially localized in the soluble fraction, the KCl concentration-dependent extractions were repeated using 1- to 2-mg pieces of the left ventricle tissue from three mice fed the D_3_-leucine Mouse Express for 5 days, being three biological replicates. In these experiments only a single extraction was performed.

To remove the relaxing solution in preparation for LC-MS, the protein within each microcentrifuge tube was precipitated by addition of 5% trichloroacetic acid (TCA). The samples were centrifuged at 18,800 RCF for 5 min, and supernatants were discarded. Each sample was resuspended in 500 μl of water and precipitated once more in 5% TCA to remove residual solution. Each sample was then resuspended in 90 μl 50 mM ammonium bicarbonate solution and digested for 18 h at 37 °C by addition of 25 μl of a 0.2 μg/μl trypsin (Promega) in 50 mM ammonium bicarbonate solution. Following digestion, 100 μl of 7% formic acid in 50 mM ammonium bicarbonate was added to inactivate trypsin, and samples were dried down by centrifugal evaporation. The samples were resuspended in TFA, centrifuged for 5 min at 18,800 RCF, and the supernatant was analyzed by LC-MS as described above.

To quantify the abundance of proteins in each fraction of samples from the three solubility assays, the resultant LC-MS Xcalibur .*raw* files were analyzed using PD 2.2 to identify the LC-MS peaks and quantify the peak areas. Searches were performed as described previously, with two differences: carbamidomethylation of cysteine was included as a variable modification of cysteine and bovine albumin (Uniprot accession P02769, downloaded 10/12/18) was included among the protein sequences. The resultant LC peak areas from these analyses were exported to Excel (supplemental Tables S3–S5). To determine the abundance of proteins in the soluble and insoluble fractions, the abundances of the top three ionizing peptides for each protein were averaged and normalized to the average abundance of the top three ionizing peptides from bovine serum albumin (supplemental Tables S6 and S7). The percentage of each protein in the soluble fraction was defined as the abundance of the protein in the first extraction, divided by the summed abundance of the protein in all the fractions. Each experimental condition was carried out with a minimum of three analytical replicates. The average ±SD was graphed using GraphPad Prism 8 and fitted with the one phase exponential where appropriate. Significant difference between rate constants and plateaus were determined using an extra sum-of-squares F test. For the experiments determining whether D3-labeled proteins were preferentially found in the soluble fraction, the abundance of unlabeled- and deuterium-labeled protein was determined from multiple peptides containing three leucine molecules (supplemental Table S8). The average abundance ±SD was graphed using GraphPad Prism 8 and differences were determined using a Student’s *t* test.

### Real-Time Visualization of Myosin Filament Depolymerization

To visualize the depolymerization of myosin within the sarcomere, two FVB mice were transfected with 100 μl of AAV. Expression of GFP-tagged RLC occurred for 12 ± 2 days prior to cervical dislocation, removal of the heart, and imaging by multiphoton microscopy. The fresh hearts were sliced open, pinned to a Sylgard (Dow Corning) coated dish to expose the papillary muscle. An aliquot of 250 mM KCl relaxing solution was added to the dish, and 250 μg/ml of saponin (Fluka Biochemika) was added to poke holes in the cellular membranes prior to onset of fluorescence imaging.

Fluorescence images were obtained through a 20× Plan Apo 1.0 NA DIC VIS-IR water immersion lens using a Zeiss LSM-7 multiphoton system. Multiphoton excitation was generated by a Coherent Chameleon Vision II Titanium Sapphire pulsed IR laser at 900 nm wavelength with dispersion compensation. The system had four nondescanned photomultiplier tube detectors, two of which make up the Zeiss gallium arsenide phosphide BiG detector. The stage was a motorized Prior Z-deck, which was fully integrated into the Zeiss Zen software. The sample was excited with 6 to 8% laser intensity. Images with 102.5 nm × 102.5 nm pixel size were collected every 30 s for 30 min after the addition of the saponin. Images were viewed in Fiji (ImageJ). Plot profiles were generated by Fiji (ImageJ) with intensity readings reported for every 102.5 nm. Measurements of the width of florescence from a single sarcomere at ½ max peak intensity were performed with manual linear interpolation from the ascending to descending legs of each peak.

## Results

### Feeding D_3_-Leucine had no Effect on Body, Heart, or Ventricular Mass or the Function of Myosin

**Fig. 2:**
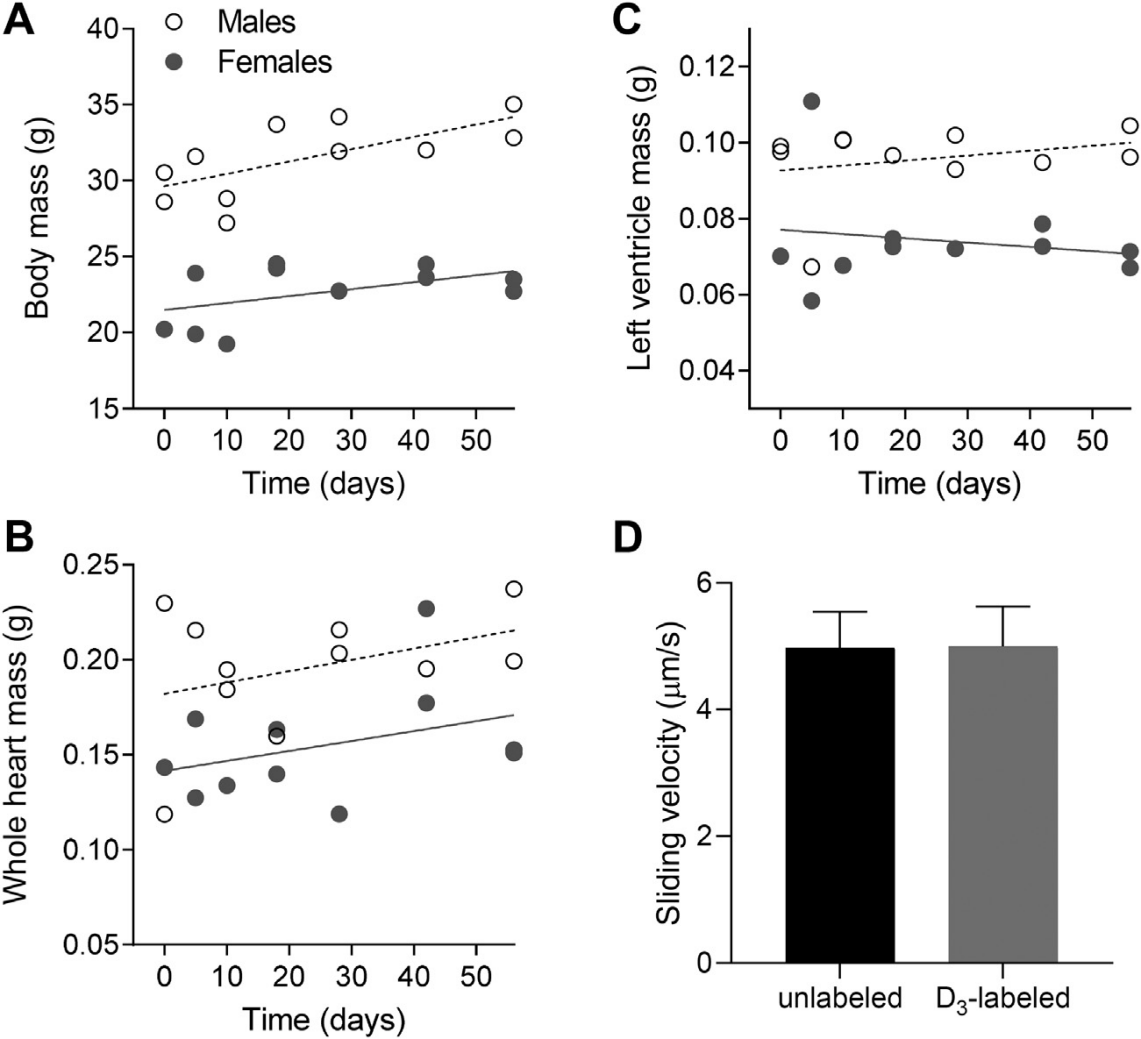
**Effect of D**_**3**_**-labeled leucine on muscle mass and myosin function during the 8-week experimental protocol.** (*A*) body, (*B*) whole-heart, and (*C*) *left ventricle* mass in 11 male (*open symbols*) and 11 female (*solid symbols*) mice. Data were fitted with linear regressions. *D*, average velocity ±SEM of bare actin filaments sliding over myosin molecules isolated from three control mice (day 0, *black*) and three mice fed D_3_-labeled leucine for 8 weeks (day 56, *gray*).

The synthesis and degradation, *i.e.*, turnover, of cardiac myosin and MyBP-C were examined in adult mice, being 3 to 5.5 months of age, by feeding a diet that contained 99% D_3_-leucine. Several measurements were made to ensure the mice were fully grown and determine whether the D_3_-leucine had physiological effects. The body mass of the females was 28% less than that of the males (*p* < 0.01), and there was only a 6% increase (*p* = 0.02) in the body mass of the male mice during the 8-week experimental protocol (Fig. 2*A*). The whole heart (Fig. 2*B*) and left ventricular masses (Fig. 2*C*) of the female mice were 21% and 22% less than that of the males (*p* < 0.01), but neither mass changed (*p* > 0.2) during the 8-week period. These data demonstrate that the mice were nearly fully grown at the start of the 8-week experimental protocol and the D_3_-leucine had no effect on whole animal mass.

To determine the impact of the D_3_-leucine on the molecular function of myosin, myosin was purified from the hearts of mice euthanized prior to feeding the diet (day 0) and at the end of the experimental protocol (day 56). The function of myosin was quantified using an *in vitro* motility assay. The velocity of fluorescently labeled actin filaments sliding over myosin did not differ (*p* = 0.5) between groups (Fig. 2*D*). Therefore, the diet did not appear to affect contractile function, as measured in this assay.

### The Appearance of D_3_-Leucine in the Intracellular Pool of Amino Acids was Governed by Two Processes

**Fig. 3:**
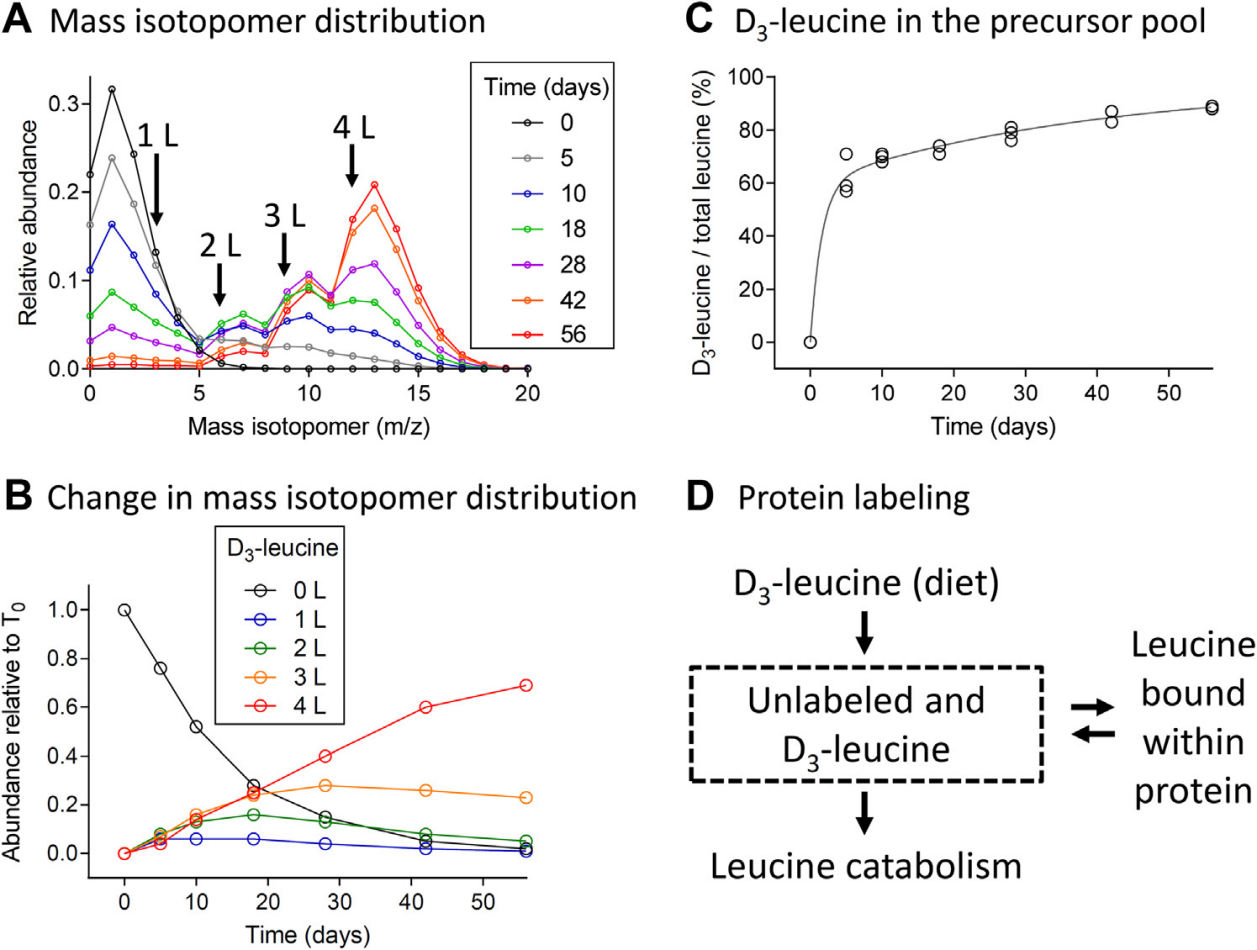
**Quantification of D**_**3**_**-leucine in the precursor pool of amino acids.***A*, relative abundance of mass isotopomers determined for a myosin heavy chain peptide that contained four potential sites of incorporation over the 8-week experimental protocol. *B*, abundance of the mass isotopomers of peptide shown in (*A*) containing 0, 1, 2, 3, and 4 labeled leucine residues during the 8-week experimental protocol. *C*, fractional abundance of D_3_-leucine in the precursor pool determined during the 8-week experimental protocol. Each point represents the average value determined from five myosin heavy chain peptides. (N = 22 mice). *D*, schematic representation of leucine entry into and exit from the precursor pool.

In the experimental diet, 99% of the leucine was D_3_-labeled. Although leucine is an essential amino acid, prior studies have demonstrated a considerable amount of amino acid recycling *in vivo* (40). It could not be assumed that the abundance of D_3_-leucine in the precursor pool immediately equaled that of the dietary enrichment. Therefore, the abundance of D_3_-leucine in the precursor pool was determined from the binomial distribution of mass isotopomers within peptides containing multiple leucine residues. Examples of the changes in mass isotopomer distribution during the 8-week experimental protocol are shown for the myosin heavy chain (shared between *MYH6/MYH7*) ALQEAHQQALDDLQAEEDKVNTLTK peptide (Fig. 3*A*). These data demonstrate that the peptide is found with 2, 3, or 4 D_3_-leucine molecules incorporated and there is a shift toward the incorporation of multiple molecules over time (Fig. 3*B*). These data were indicative of an increase in the abundance of D_3_-leucine in the precursor pool with respect to time.

The amount of D_3_-leucine relative to all of the leucine in the precursor pool during the 8-week experimental protocol (Fig. 3*C*) was determined from the binomial probability of incorporating D_3_-leucine into five α-myosin heavy chain peptides, each containing 3 to 4 leucine residues, using mass isotopomer distribution analysis (39, 40). The change in abundance of the D_3_-leucine in the precursor pool was well fitted (R^2^ = 0.994) by a double exponential equation (Fig. 3*C*). The initial rate constant (k = 0.64 ± 0.21) was 21 times faster than that of the second rate constant (k = 0.03 ± 0.01 days^−1^) and the plateau of the equation was 98% (Fig. 3*B*), being similar to the 99% expected from the D_3_-leucine diet. There was a large degree of variability in precursor labeling at the early time points (*e.g.*, 19% (SD) at day 5), which diminished to 2% (SD) by Day 56 of the experiment. This variability may result from diurnal oscillations in amino acid availability that become less apparent over time (40).

### Unlabeled Myosin Heavy Chain, Myosin Light Chain, and MyBP-C Were Replaced by Newly synthesized Molecules at Similar Rates

**Fig. 4:**
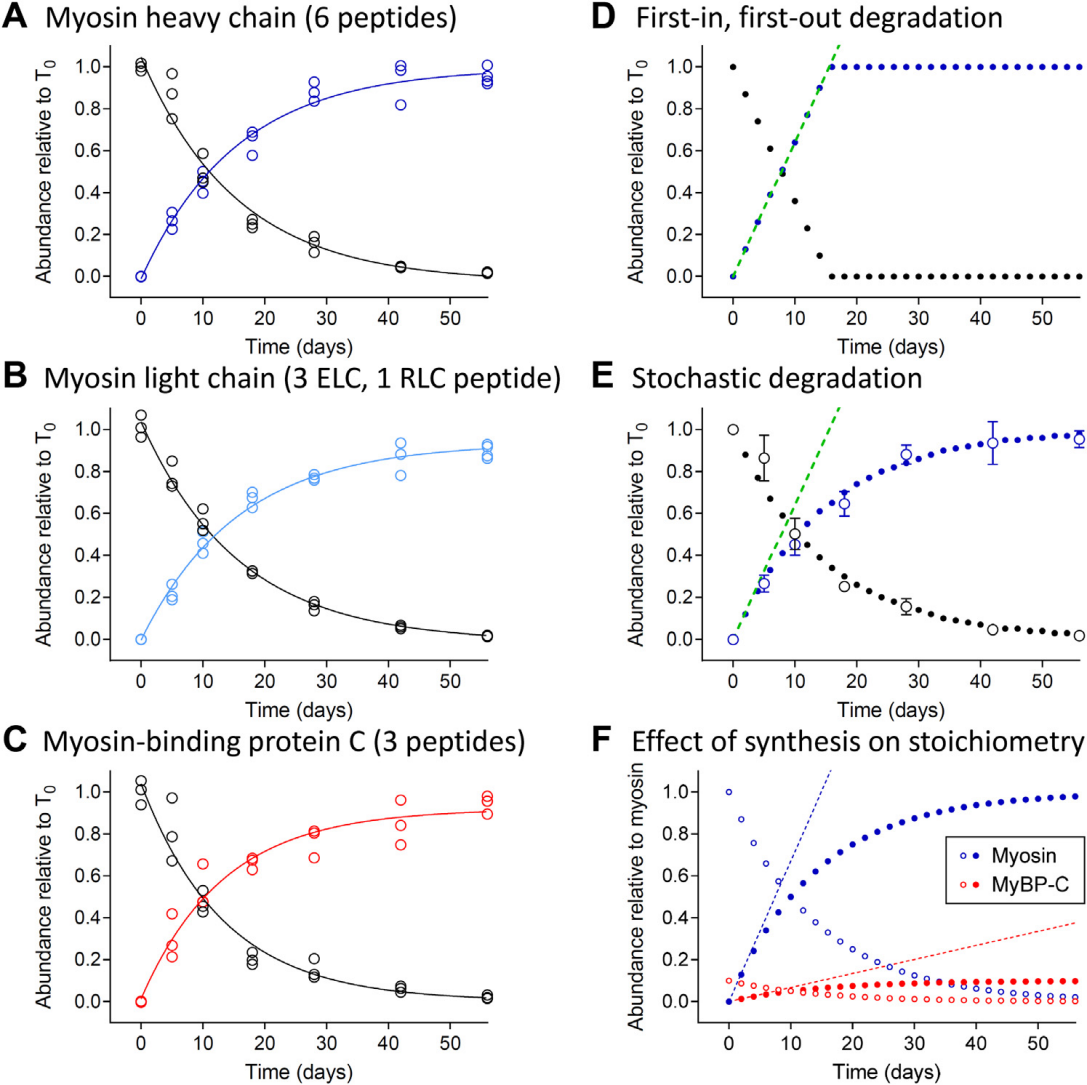
**Quantification of thick filament protein turnover in the apex of the left ventricle and interpretation through modeling.** The abundance of unlabeled (*black*) and deuterium-labeled (*A*) myosin heavy chain (*dark blue*), (*B*) myosin *light chain* (*light blue*), and (*C*) MyBP-C (*red*) during the 8-week course of the experimental protocol. The data comprise pairs of data points (unlabeled and deuterium labeled) for each mouse used in the study (N = 22 mice). The data were fitted with one phase decay curves to compare the rate constants and half-lives. Analytical models developed to demonstrate the change in the abundance of unlabeled (*solid black*) and deuterium-labeled (*solid blue*) protein, when (*D*) protein synthesis (*dashed green line*) and degradation follow a first-in, first-out, zero-order kinetic scheme and (*E*) protein synthesis follows a zero-order kinetic scheme (*dashed green line*) while degradation is first order and thus dependent on the concentration of each molecule. The turnover of myosin (*open symbols*) is well described by (*E*), the model with stochastic degradation. *F*, analytical model demonstrating the impact of the rate of myosin (*dashed blue line*) and MyBP-C (*dashed red line*) synthesis on the relative abundance of the two proteins.

To determine the abundance of unlabeled- and deuterium-labeled myosin molecules within each heart, we quantified mass isotopomer abundances for six α-myosin heavy chain peptides, each containing 2 to 4 leucine residues. We corrected the mass isotopomer distribution for each peptide to account for naturally occurring isotopes and the degree of precursor labeling and normalized the values relative to the total protein in each sample. The mean abundances of the molecules translated prior to the start of feeding the D_3_-leucine (unlabeled protein) and translated after the introduction of the D_3_-leucine (deuterium-labeled protein) were plotted for each mouse (Fig. 4*A*).

The changes in the mean abundance of the unlabeled and deuterium-labeled myosin heavy chain molecules (Fig. 4*A*) were well fitted with a one phase exponential (R^2^ > 0.97) with the asymptotes being 0.00 ± 0.03 and 0.99 ± 0.03, respectively. The asymptotes demonstrated that nearly all of the unlabeled myosin molecules were replaced with labeled molecules during the 8-week course of the experimental protocol and the total abundance of the protein was unchanged. The rate of change in the abundance of the unlabeled and deuterium-labeled molecules, being 0.065 ± 0.004 days^−1^ was shared between the two curves (*p* = 0.70). These data were indicative of the unlabeled and deuterium-labeled myosin having identical 10.8-day half-lives.

To determine whether the changes in abundance of the myosin light chains or MyBP-C differed from that of the heavy chain, the mass isotopomer abundances were quantified for four myosin light chain (three from *MYL3* and one from *MYL2*) (Fig. 4*B*) and three MyBP-C (*MYBPC3*) peptides (Fig. 4*C*) using an identical procedure. The rates of change in the abundances of the unlabeled and deuterium-labeled proteins, being 0.065 ± 0.004 and 0.076 ± 0.007 days^−1^, respectively, were similar to that of the myosin heavy chain molecules (Fig. 4*A*). The total abundance of myosin was 10.3 ± 0.7 (SD) times that of MyBP-C in the day 0 sample, as determined from the average abundance of the top three ionizing peptides for each protein. However, the rates of change in abundance of the unlabeled and deuterium-labeled proteins are shared between myosin heavy chain, myosin light chain, and MyBP-C in the heart under normal conditions.

### The Degradation of Thick Filament Proteins Occurs *via* a Stochastic Mechanism

To provide mechanistic insight into the rates of change in the abundance of the unlabeled and deuterium-labeled proteins, two analytical models were developed. Both models assumed that protein synthesis occurred *via* a zero-order process (green dashed line in Fig. 4, *D* and *E*), with a constant number of molecules being added to the muscle with respect to time. To maintain total protein abundances, both models assumed the number of molecules removed at each time point was equimolar to those added, as observed over the 8-week experimental protocol (Fig. 4, *D* and *E*). The two models differed in how the molecules were selected for degradation. The first model (Fig. 4*D*) assumed that there was a time dependence to the selection of the molecules, with those first present in the system given preference for degradation. This reflected a first-in, first-out type mechanism of degradation and resulted in linear changes in the abundances of the unlabeled and labeled molecules (Fig. 4*D*). The second model (Fig. 4*E*) assumed the molecules were selected for degradation by a first-order, concentration-dependent mechanism with no preference for older molecules. This resulted in an exponential decrease in the abundance of the unlabeled molecules and reciprocal increase in the abundance of the labeled molecules (Fig. 4*E*). The changes in abundances of the proteins in the experimental data were much more similar to those described by the second model (Fig. 4*E*). At day 10.5 in this model, the concentration of unlabeled molecules was equal to that of the labeled molecules, and their likelihood of selection for degradation was equivalent.

### Protein Synthesis Determines Thick Filament Protein Stoichiometry

While the rates of degradation of myosin and MyBP-C were equivalent (Fig. 4, *A*–*C*), myosin was 10.3 ± 0.7 (SD) times more abundant than MyBP-C in the heart samples at day 0, as determined from the average abundance of the top three ionizing peptides for each protein. The relative abundance of these proteins did not appear to change during the 8-week experimental protocol (Fig. 4*C*). To account for the difference in the abundance, we modified the analytical model. When the rate of MyBP-C synthesis was reduced ∼10-fold in comparison with that of myosin, the ∼10-fold difference in stoichiometry was conserved across all time points (Fig. 4*F*). These data demonstrate that the ∼10 to 1 difference in stoichiometry between myosin and MyBP-C is determined by the rate of protein synthesis under normal conditions required for cellular maintenance.

### Myosin and MyBP-C Molecules Exist in an Equilibrium Between Thick Filaments and a Soluble Pool of Protein

**Fig. 5:**
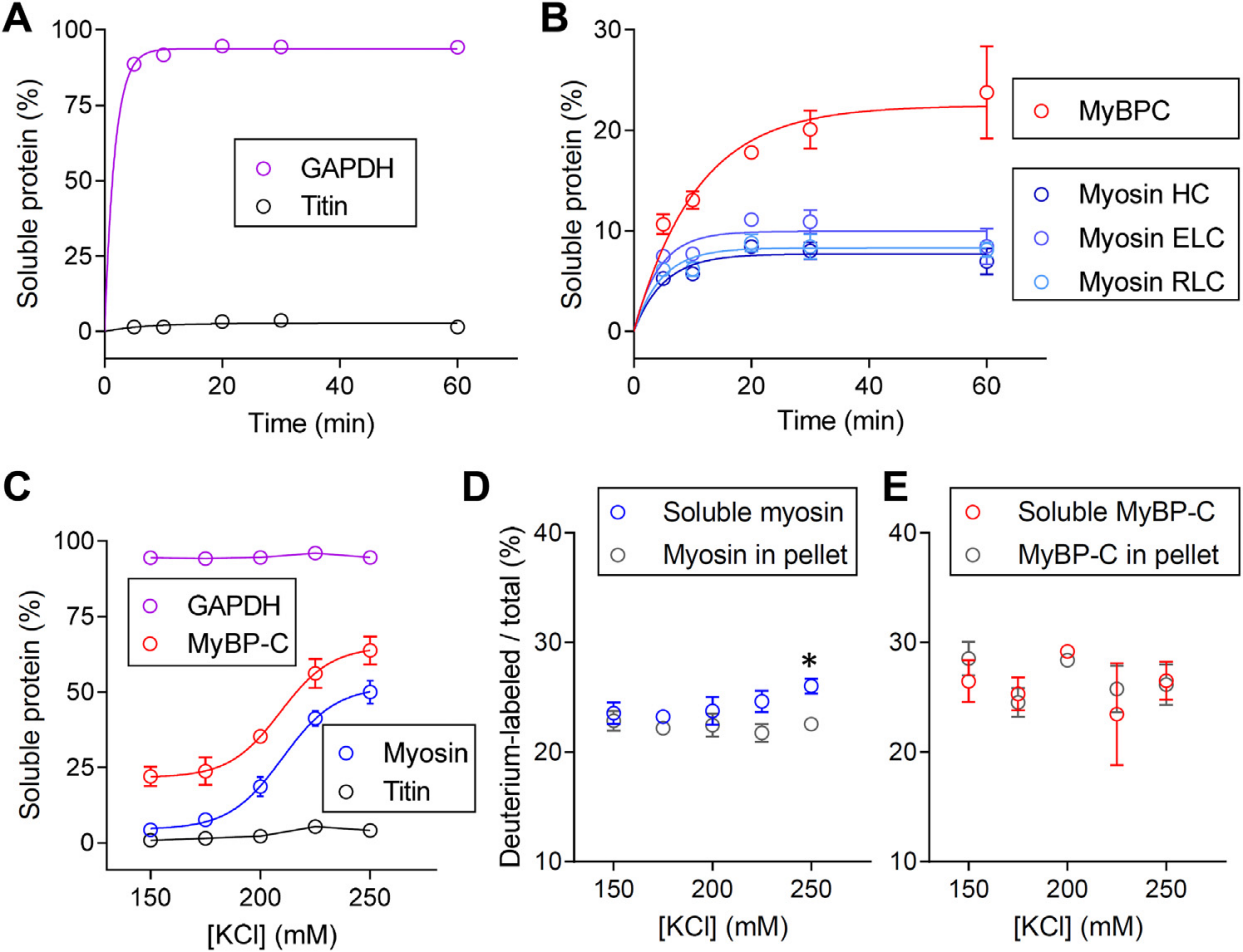
**Biochemical evidence for the rapid mixing of myosin molecules within thick filaments in the intact heart.** The time-dependent solubility of (*A*) GAPDH and titin, and (*B*) the *thick filament* proteins when cardiac muscle was equilibrated to 175 mM KCl extraction buffer containing 0.1% Triton-X 100 to permeabilize cellular membranes. *C*, the ionic strength-dependent solubility of the thick filament proteins, GAPDH, and titin when exposed to extraction buffer for 60 min. The ionic strength-dependent distribution of deuterium-labeled (*D*) myosin and (*E*) MyBP-C in the soluble fraction and pellet when muscle from the minimally labeled day 5 samples was exposed to extraction buffer for 60 min ∗*p* < 0.01.

To determine whether newly synthesized molecules are assembled into new thick filaments or mixed into preexisting filaments, thick filament proteins were extracted from the muscle. First, the time-dependent stability of the sarcomere was determined by removing the cellular membrane with Triton X-100 and exposing the muscle to 175 mM KCl relaxing solution for finite periods. The skinned samples were separated into soluble and insoluble fractions, and the composition of each fraction was quantified by LC-MS. These initial experiments demonstrated that nearly 100% of the cytosolic protein, glyceraldehyde 3-phosphate dehydrogenase, was rapidly released into the extraction buffer. In contrast, nearly 100% of titin remained in the insoluble fraction for the 60-min duration of the experiment (Fig. 5*A*). These data demonstrate the samples can be precisely separated into two specific fractions containing soluble and insoluble proteins.

Myosin heavy chain, essential light chain, and RLC were rapidly released into the extraction buffer, and there was no apparent difference in the rates of release or maximal amount of each protein extracted at any time point (Fig. 5*B*). The similarity in extraction kinetics suggests the components of the myosin molecules are tightly coupled *in vivo* and are coextracted from the muscle. In contrast, the rate of MyBP-C release appeared to differ from that of myosin and the maximal amount of MyBP-C appeared to be greater (Fig. 5*B*). The difference in the extraction kinetics between myosin and MyBP-C suggests the proteins found in the extraction buffer are depolymerized molecules rather than thick filaments.

Next, a second set of experiments were performed using 60-min extractions over a range of conditions to examine the effect of ionic strength on protein extraction (Fig. 5*C*). Nearly 50% of the thick filament proteins were solubilized at the highest ionic strength (250 mM KCl) tested (Fig. 5*C*). The abundance of MyBP-C found in the soluble fraction was greater than myosin, particularly at the lower ionic strengths examined (Fig. 5*C*). The differences in myosin and MyBP-C abundances in the cytosol, even under the weakest ionic conditions tested, suggested that individual molecules are found in the cytosol rather than intact thick filaments.

These extraction experiments were repeated with heart tissue from the mice that were fed the D_3_-leucine diet for 5 days. At this time point 25 ± 3% (SD) of the thick filament proteins were labeled with deuterium (Fig. 4, *A*–*C*). The abundance of the deuterium-labeled molecules relative to all the molecules was quantified in the soluble and insoluble fractions. The relative abundance of labeled and unlabeled myosin was identical between fractions at low salt concentrations. There was a slight difference (*p* < 0.01) in the abundance of labeled myosin in the soluble fraction at the highest ionic strength (Fig. 5*D*), where ∼50% of the myosin had been depolymerized (Fig. 5*C*). There was no difference in the abundance of the labeled and unlabeled MyBP-C between the two fractions (Fig. 5*E*). These data suggest that the newly synthesized proteins are not sequestered within the cytosol and are somewhat randomly mixed within thick filaments in the intact heart within 5 days.

### Myosin Molecules at the Tips of the Filaments Depolymerize Rapidly

**Fig. 6:**
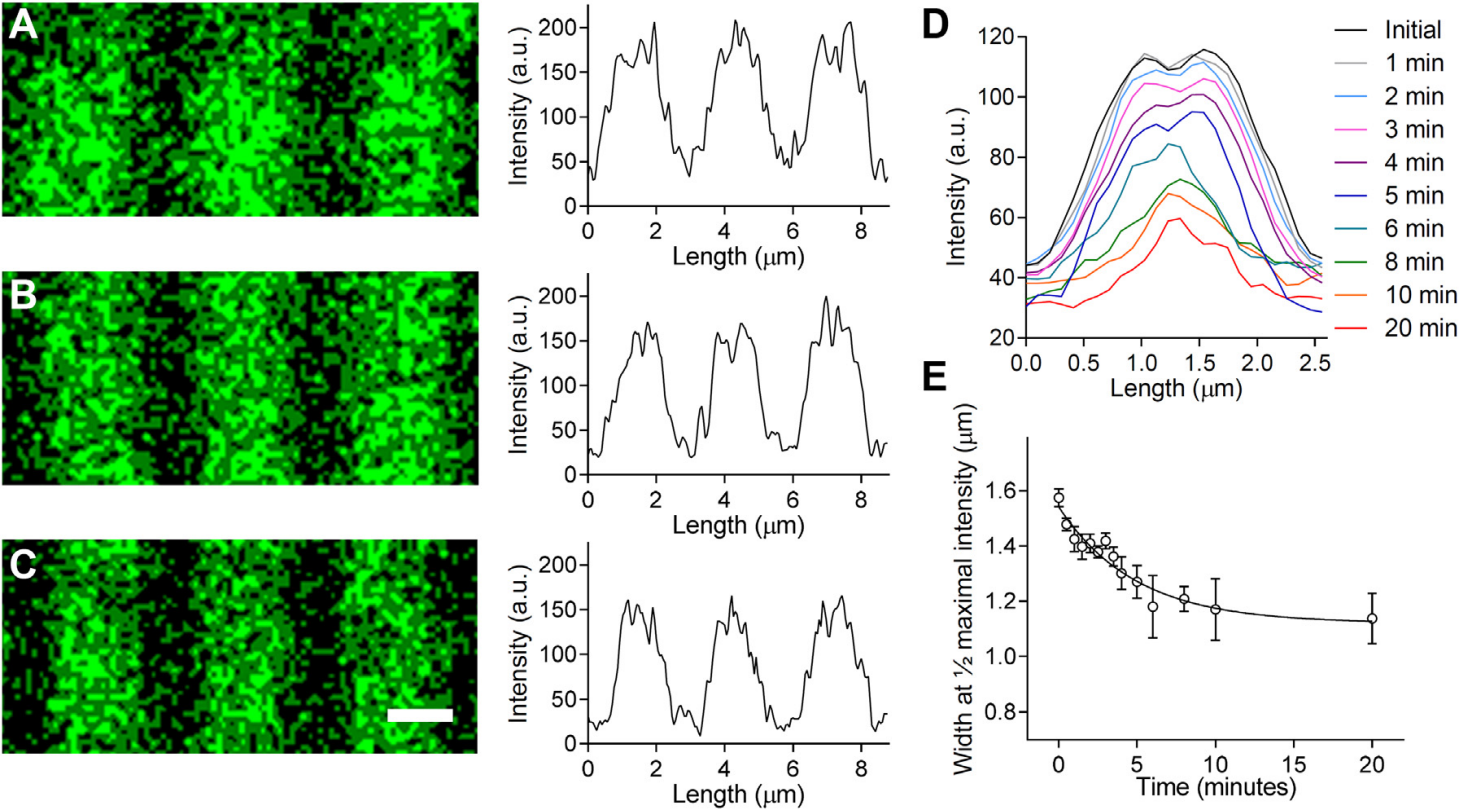
**Visualization of the depolymerization of GFP-labeled myosin from thick filaments within cardiac muscle sarcomeres exposed to 250 mM KCl.** Fluorescence images and intensity plot profiles of three adjacent sarcomeres in a papillary muscle (*A*) prior to skinning and (*B*) 1.5 min and (*C*) 3 min after exposure to 250 mM KCl. The scale bar represents 1 μm. *D*, aligned and averaged plot profiles for sarcomeres imaged at 1-min intervals following exposure to 250 mM KCl in two heart preparations. (N = 20 sarcomeres per time point). *E*, width of florescence in each plot profile at ½ maximal intensity with respect to time fitted with a one phase exponential decay.

To visualize the depolymerization of myosin molecules from the filaments, mice were transfected with an AAV that induced the expression of myosin regulatory light chain with green fluorescent protein tag on the C terminus. Bands of fluorescence were observed in the intact muscle with multiphoton imaging (Fig. 6*A*), each being consistent with the length of the thick filaments within single sarcomeres (Fig. 1*A*). When the fluorescence profiles from such bands (Fig. 6, *A*–*C*) were aligned and averaged (Fig. 6*D*), a dip in fluorescence was observed in the center of the band, where the thick filaments are devoid of myosin heads (Fig. 1*A*). Upon disruption of the cellular membrane and exposure of the sarcomere to the 250 mM KCl, the width of these bands at ½ maximum fluorescence intensity was rapidly reduced (Fig. 6, *D* and *E*), as myosin molecules were presumably released into the soluble fraction (Fig. 5*C*). This decrease in length of the fluorescence profile demonstrated that molecules were preferentially lost from the tips of the filaments during depolymerization rather than being evenly lost along the filament length.

## Discussion

Advancements in the preservation and imaging of both thick filaments and muscle tissue have provided exceptional insight into the exquisite organization of striated muscle myosin and MyBP-C molecules (4, 5, 9). It is well established that thick filament structure is critical for the binding of myosin molecules to actin filaments to generate contractile forces. However, there is a large gap in our knowledge of how muscle maintains this organization while replacing its molecular constituents through protein synthesis and degradation (22, 23, 24, 27). Therefore, our aim was to evaluate the temporal and spatial mechanism by which thick filament proteins are replaced in the hearts of adult mice under steady-state conditions.

To address this aim we fed mice a diet in which 99% of the leucine was labeled with deuterium (D_3_-leucine), and we quantified the appearance of the D_3_-leucine within both the precursor pool of amino acids required for protein assembly and within newly synthesized thick filament proteins. The diet had no discernable effects on body, whole heart, or ventricular masses over the 8-week period (Fig. 2, *A*–*C*), nor did the incorporation of the D_3_-leucine impact the molecular function of myosin (Fig. 2*D*). These data demonstrate that the D_3_-leucine-labeled diet is palatable for mice and does not perturb whole animal, muscle, or molecular physiology.

**Fig S1:**
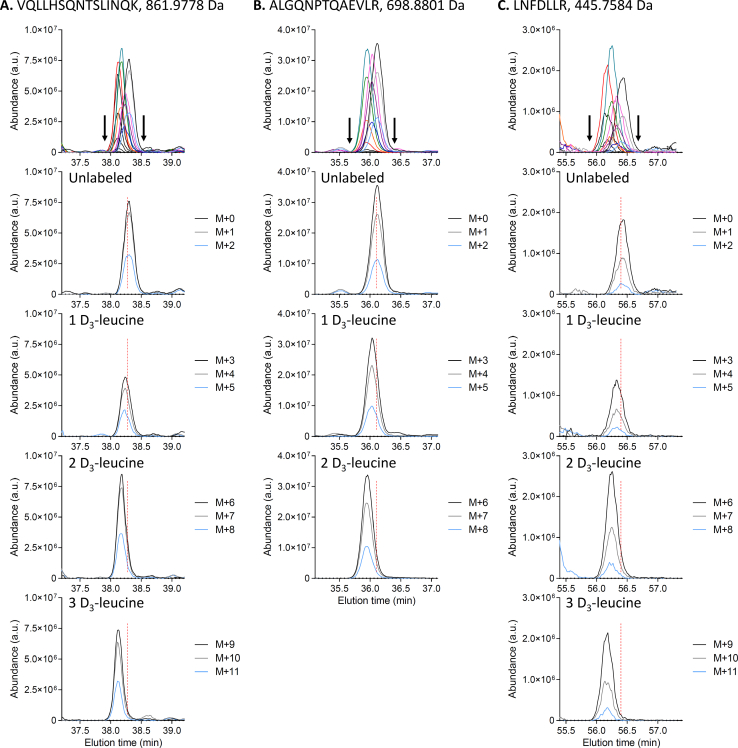
LC elution peak profiles for individual mass isotopomers of peptides from (*A*) myosin heavy chain, (*B*) myosin essential light chain, and (*C*) MyBP-C present in a sample from Day 18. Top panels show all LC elution profiles for each isotopomer and the start and stop points used for integration. Lower panels show LC elution profiles for isotopomers for peptides with 0, 1, 2, and 3 D_3_-leucine residues incorporated. Red dashed lines are indicative of the LC elution time for the mass isotopomers containing 0 D_3_-leucine residues. There is a leftward shift in the LC elution profile corresponding to the number of D_3_-leucine residues incorporated into each peptide.

Previous studies labeling mammals with valine and lysine (40, 47) have demonstrated that there is a considerable amount of recycling of amino acids within the precursor pool, which must be accounted for when examining protein turnover. The use of a diet containing 99% D_3_-labeled leucine provides a significant advantage over other labeling strategies, because many tryptic peptides contain multiple leucine residues, and the distribution of labeled molecules in these peptides (Fig. 3*B*) can be used to quantify the precursor pool labeling (Fig. 3*C*) by mass isotopomer distribution analysis (39). However, care must be taken for the analyses, as many mass isotopomers overlap in the mass spectra (Fig. 3*A*), and there is a significant amount of shift in the LC retention time between these mass isotopomers (supplemental Fig. S1).

The rate of appearance of D_3_-leucine in the precursor pool was well described by a double exponential equation (Fig. 3*C*). We interpret this finding to indicate that the D_3_-leucine ingested from the diet was continually mixed with leucine molecules recycled from the breakdown of preexisting proteins (Fig. 3*D*). The rapid increase in the appearance of the D_3_-leucine in the precursor pool suggests that much of the pool of amino acids is derived from the diet. However, the slower rate of appearance demonstrated that this pool is continually being diluted with amino acids released from degraded proteins and a fraction of these will get reincorporated into newly synthesized proteins, escaping catabolism. Not accounting for this recycling when using labeled amino acids with only a single site of incorporation into a peptide would induce a global error in protein synthesis and degradation calculations.

A second advantage of the use of D_3_-labeled leucine as a precursor label is the ability to obviate errors associated with the lack of instantaneous labeling of the precursor pool by only evaluating the abundances of peptides containing two or more leucine residues for synthesis and degradation calculations. This approach enhanced the probability that at least one D_3_-leucine molecule was incorporated into every tryptic peptide used for analysis. This minimized the need for corrections to quantify the abundance of the unlabeled and labeled proteins.

The rates of changes in the abundance of the unlabeled and labeled myosin heavy chain, myosin light chain, and MyBP-C molecules within the heart were nearly identical, with 50% of each protein being replaced in 10.8 days (Fig. 4, *A*–*C*). These data were well described by an analytical model in which a constant amount of each protein was synthesized, and an equivalent fraction of each protein was degraded with respect to time (Fig. 4*E*). Thus, newly synthesized molecules of each protein had an equal probability of being degraded to older molecules once ∼50% of the protein pool was replaced (Fig. 4*E*). Owing to this balance, the total protein abundances were constant during the 8-week experimental protocol (Fig. 4, *A*–*C*). Therefore, the 10.3 ± 0.7 (SD) to 1 difference in stoichiometry of myosin to MyBP-C was maintained. This ∼10-fold difference in stoichiometry must result in a ∼10-fold difference in the amount of each protein synthesized per unit time (Fig. 4*F*), because the fraction of each pool degraded was nearly identical. These data suggest that the rate of thick filament protein synthesis defines the molecular composition of the filaments under normal conditions required for cellular homeostasis.

With respect to the balance between protein synthesis and degradation, recent results from our laboratory demonstrated that heterozygous truncation mutations in the *MYBPC3* gene, which cause hypertrophic cardiomyopathy, reduced the abundance of full-length *MYBPC3* mRNA and the rate of MyBP-C synthesis in human induced pluripotent stem cells (48). Based on our modeling (Fig. 4*E*), this defect in protein synthesis should have resulted in a reduction in MyBP-C content, as observed in adult human myocardium (43, 49, 50). However, in the induced pluripotent stem cells, MyBP-C content was preserved by blunting the rate of MyBP-C degradation (48). Our current data suggest that the previous finding (48) may be specific to the IPSCs under the culture conditions examined and limited timeframe of the observations. Additional studies are needed to understand the role of MyBP-C content in the modulation of thick filament protein degradation *in vivo* to gain insight into the complex role of proteostasis in diseases such as hypertrophic cardiomyopathy.

The more general observation that thick filament protein degradation occurred *via* a stochastic mechanism could be explained by competing hypotheses that thick filaments are stochastically degraded or that individual molecules are in an equilibrium with the thick filaments and randomly selected for degradation. To test these hypotheses, thick filament proteins were extracted from muscle under a range of conditions that disrupted the ionic interactions that hold the filaments together (51). The three components of a single myosin molecule, being the heavy chain and the essential and regulatory light chains, were coextracted from the heart tissue (Fig. 5*B*) under all ionic strengths tested (Fig. 5*C*). Both the rate at which MyBP-C was extracted (Fig. 5*B*) and the total amount of MyBP-C extracted (Fig. 5*C*) appeared to differ from that of the myosin molecules. These data suggested that myosin and MyBP-C have different disassociation kinetics and affinities for the thick filament macromolecular complex, as supported by earlier studies (51). The lack of similarity in the abundances of the extracted myosin and MyBP-C molecules, even at the lowest ionic strengths tested, suggests that sarcomeres contain a pool of single molecules rather than a pool of easily releasable thick filaments, as previously proposed (30, 31, 32).

In contrast to these findings, the observation that all three components of a myosin molecule were coextracted from the muscle (Fig. 5*B*) and have similar rates of turnover (Fig. 4, *A* and *B*) allows for the hypothesis that the myosin molecule exists as a unit *in vivo* and its components are replaced together. This hypothesis is supported by findings that that association of myosin heavy chain and light chains occurs early after translation, even prior to the complete folding of the molecule (52). Retention of the light chains on the heavy chains may be essential to ensure proper contractile function (53). However, the hypothesis is at odds with previous results from rat hearts (54, 55) and chicken embryonic cell culture systems (22) that demonstrate the components of myosin have different turnover kinetics. This discrepancy may be due to the improvement of the current analytical techniques over those initially used to make these measurements.

To test whether the newly synthesized molecules were organized into new thick filaments or incorporated into the preexisting structures, the extraction experiments were performed using the samples from the day 5 mice that contained minimal labeling. The relative distribution of the unlabeled and deuterium-labeled thick filament proteins was similar between the fractions, with the exception of myosin in the samples exposed to 225 and 250 mM KCl (Fig. 5*D*). In these samples, there was a slight bias in the amount of labeled myosin in the soluble fraction. To determine whether this bias could result from there being more exchange of myosin molecules at the tips of the thick filaments, myosin was labeled with GFP *in vivo* and the extraction process was visualized in real-time with multiphoton microscopy (Fig. 6, *A*–*E*). The change in the florescence profile across the length of single sarcomeres demonstrated that the myosin molecules were preferentially extracted from the thick filaments, as described previously for skeletal muscle (56, 57). The total amount of myosin extracted (Fig. 6*D*) and kinetics of the extraction (Fig. 6*E*) were similar to that observed in the biochemical assays (Fig. 5, *B* and *C*). The preferential removal of myosin molecules from the tips of the thick filament combined with the bias in D_3_-labeling in this region suggests that the exchange of molecules at the ends of the thick filaments may be more dynamic than near the center. Such rapid mixing of molecules into the tips of the thick filaments may be critical for the rapid sequestration of molecules into filaments after they are synthesized.

Thick filaments are most often visualized as rigid structures (4, 5, 9) with the sole responsibility of bearing molecular forces required for muscle contraction. However, our data suggest thick filaments are dynamic macromolecular complexes with an intrinsic functionality that allows for the replacement of molecules while supporting muscle contractility. These findings of stochastic replacement are in contrast to hypotheses regarding protein turnover in other areas such as the plant photosynthesis field (58). While it is compelling that photodamage triggers the replacement of proteins from large complexes, this has been harder to prove. This replacement may be stochastic, with the proteins being rapidly turned over on a regular basis as a part of intracellular maintenance. Future studies should be aimed at elucidating the timing and mechanisms by which myosin molecules are exchanged into and out of thick filaments to determine whether this relates to the rate at which the proteins are replaced. It is possible that perturbations in the rates of exchange affect the availability of the molecules for protein replacement (Fig. 1*B*) and play a role in cardiac diseases that are characterized by changes in muscle mass or decrements in function, such as hypertrophic cardiomyopathy.

## Data Availability

The Thermo Xcalibur .raw files and PD 2.2.pdresult files have been deposited to MassIVE (https://massive.ucsd.edu; Dataset MSV000088817). The data files are robust with information regarding the turnover of many cardiac proteins. We hope that groups interested in the turnover of proteins within other subcellular organelles, such as mitochondria, take advantage of these data files.

## Supplemental data

This article contains supplemental data.

## Conflict of interest

The authors declare that they have no conflicts of interest with the contents of this article.
